# Cost-effectiveness of monitoring glaucoma patients in shared care: an economic evaluation alongside a randomized controlled trial

**DOI:** 10.1186/1472-6963-10-312

**Published:** 2010-11-17

**Authors:** Kim M Holtzer-Goor, Esther van Sprundel, Hans G Lemij, Thomas Plochg, Niek S Klazinga, Marc A Koopmanschap

**Affiliations:** 1 Institute for Medical Technology Assessment-Erasmus University Rotterdam, Rotterdam, the Netherlands; 2 Rotterdam Ophthalmic Institute, Rotterdam, the Netherlands; 3 The Rotterdam Eye Hospital, Rotterdam, the Netherlands; 4 Department of Social Medicine, Academic Medical Center (AMC)/University of Amsterdam, the Netherlands

## Abstract

**Background:**

Population aging increases the number of glaucoma patients which leads to higher workloads of glaucoma specialists. If stable glaucoma patients were monitored by optometrists and ophthalmic technicians in a glaucoma follow-up unit (GFU) rather than by glaucoma specialists, the specialists' workload and waiting lists might be reduced.

We compared costs and quality of care at the GFU with those of usual care by glaucoma specialists in the Rotterdam Eye Hospital (REH) in a 30-month randomized clinical trial. Because quality of care turned out to be similar, we focus here on the costs.

**Methods:**

Stable glaucoma patients were randomized between the GFU and the glaucoma specialist group. Costs per patient year were calculated from four perspectives: those of patients, the Rotterdam Eye Hospital (REH), Dutch healthcare system, and society. The outcome measures were: compliance to the protocol; patient satisfaction; stability according to the practitioner; mean difference in IOP; results of the examinations; and number of treatment changes.

**Results:**

Baseline characteristics (such as age, intraocular pressure and target pressure) were comparable between the GFU group (n = 410) and the glaucoma specialist group (n = 405).

Despite a higher number of visits per year, mean hospital costs per patient year were lower in the GFU group (€139 vs. €161). Patients' time and travel costs were similar. Healthcare costs were significantly lower for the GFU group (€230 vs. €251), as were societal costs (€310 vs. €339) (p < 0.01). Bootstrap-, sensitivity- and scenario-analyses showed that the costs were robust when varying hospital policy and the duration of visits and tests.

**Conclusion:**

We conclude that this GFU is cost-effective and deserves to be considered for implementation in other hospitals.

## Background

Glaucoma is a group of eye diseases characterized by damage to the optic nerve that causes gradual, irreversible visual field loss. It is often related to high intraocular pressure (IOP) and age. Usual care for glaucoma patients consists of diagnosis, lifelong monitoring and treatment, and in most countries is currently provided by glaucoma specialists.

Ophthalmic care in the Netherlands is currently being challenged by a high workload for glaucoma specialists and long waiting lists. Due to ageing of the population, the prevalence of glaucoma probably will increase strongly over time [1], possibly endangering access to glaucoma care as currently provided. Task substitution may be one way to ease this problem.

Stable glaucoma patients and patients with a risk factor for developing glaucoma may not require care by a glaucoma specialist. Instead, monitoring by hospital optometrists or ophthalmic technicians may be sufficient. This would leave glaucoma specialists with more time for complex cases and new glaucoma patients, allocating their expertise more efficiently, and also reducing waiting lists. As optometrists and ophthalmic technicians are less expensive per hour than specialists, such task substitution might save costs.

To date, only one study [2-6] presented information about the efficiency of substitution in glaucoma care, and its consequences for both quality of care and cost-effectiveness. However, that study compared care by glaucoma specialists with that by community optometrists rather than hospital optometrists. It concluded that glaucoma monitoring by community optometrists is effective, but not cost-effective in most situations. The outcomes were similar to those in specialist care, and the patients were satisfied. However, because of a (standard) shorter follow-up interval than in specialist care, community monitoring was more expensive [6].

We therefore conducted a randomized controlled trial (RCT) to determine the cost-effectiveness of shared care in stable glaucoma patients in a hospital setting. We compared usual care by glaucoma specialists and the care provided by optometrists and ophthalmic technicians within a glaucoma follow-up unit (GFU) in the Rotterdam Eye Hospital (REH) in terms of costs and quality of care.

Because this paper focuses on the costs of the glaucoma care related to important aspects of the quality of care, we also measured patient satisfaction, the number of treatment changes, the change in IOP and the compliance to the standard working protocol. The quality of care is described in more detail elsewhere. [7].

## Methods

### Randomized Controlled Trial

Patients who visited a glaucoma specialist or the GFU between September 2005 and April 2006 were invited to participate. The RCT was explained and written information was provided to them. The study was approved by the Review Board of Erasmus MC.

To be eligible for the study, patients had to meet the following criteria:

(1) the patient was diagnosed with stable glaucoma in one or both eyes (the next visit scheduled in 6 months or more) or had a risk factor for glaucoma, i.e. high IOP and/or a positive family history. Eyes were considered to be glaucomatous if they had typical thinning or notching of the neuroretinal rim of the optic nerve head, with or without disc haemorrhages, visual field defects, peripapillary atrophy and/or and elevated IOP;

(2) a glaucoma specialist of the REH referred the patient to the GFU;

(3) the actual ophthalmic medication and the target pressure (TP) was recorded in the medical record. The target pressure was determined by the individual clinicians in all patients, where they took in consideration: the age of the patient, the appearance of the optic disc, the level of intraocular pressure, any co-morbidity and any other risk factors. For patients with a risk factor for glaucoma, the TP was by default 30 mmHg, unless other risk factors called for an explicitly lower TP;

(4) an examination of the optic disc, macula, and the fundus periphery was performed;

(5) the Snellen visual acuity in each eye was ≥ 20/100 and/or the patient had no visual field loss within the central 10°, as measured by a Humphrey Field Analyser, standard 24-2 test algorithm (HFA 24-2; Carl Zeiss Meditec, Dublin, CA, USA);

(6) the refractive error was between +5 and -8 dioptres (spherical equivalent);

(7) no other significant ocular disease was present;

(8) the patient had not undergone laser therapy for diabetic retinopathy.

Once the glaucoma specialist decided that the patient was suitable for the GFU, the patient was randomly allocated to a treatment group. In the glaucoma specialist group, the patients received care of glaucoma specialists and residents only. In the GFU group the patient visited the GFU twice followed by a visit to the glaucoma specialist or resident if the patient was stable. If necessary, the patient was seen by a glaucoma specialist earlier. The GFU employees (optometrist or ophthalmic technician level 1 or 2) provided care according to a standard working protocol (see Table 1) and under supervision of glaucoma specialists.

**Table 1:** Provided care and criteria for back referral to the glaucoma specialist

Activity	GFU	Usual care	Criteria for back referral
Short history	Every visit	Every visit	
IOP*	Every visit	Every visit	
Medical prescriptions	Every visit	Every visit	
Optic disc assessment	Never	Every visit	
GDx ECC**	Every visit	At doctor's request (approx. once yearly)	- Suspicion of progression - In case of first GDxECC: NFI > 35 and/or left/right asymmetry and/or local defect.
HFA 24-2***	Yearly in moderate to advanced visual field damage**** OR at doctor's request	At doctor's request (approx. once yearly)	Suspicion of progression
Snellen visual acuity	Every visit	As required, at least once yearly	Decline in visual acuity of ≥ 2 lines
Overall judgement	Every visit	Every visit	
Timing next appointment	Every visit	Every visit	

* IOP by Goldmann applanation tonometry

** GDx ECC (Carl Zeiss Meditec, Dublin, CA, USA) scanning laser polarimetric images

*** Humphrey Field Analyser, standard 24-2 test algorithm (HFA 24-2; Carl Zeiss Meditec, Dublin, CA, USA)

**** Criteria mild and moderate/severe visual field damage: the mean deviation (MD) of the last performed visual field was ≤ -5dB

### Identification and Randomisation

Glaucoma specialists were asked to provide information about this study to eligible patients. Eligible patients were also identified by searching the patient files of the patients that were already referred by the glaucoma specialists to the GFU in the months preceding the start of our study. They received information about the study during their next visit. All patients that were eligible and willing to participate were randomly allocated to a treatment group using a randomisation table. For the GFU patients that were allocated to the usual care group an appointment was made with the glaucoma specialist who referred them to the GFU.

To avoid glaucoma specialists influencing the allocation of patients, we used central randomisation by the researchers using stratification by 2 variables: the referring glaucoma specialist, and the time to the next scheduled visit, being either 6 months or more than 6 months.

### Outcome measures

The outcome of the treatment was measured every visit. The outcome measures of the RCT were: 1) compliance of the GFU employees to the standard working protocol, 2) patient satisfaction with the following items: a) overall mark for the received care; b) social interaction with the health care provider; c) expectations about the visit; d) perceived knowledge of the health care provider; e) waiting area, 3) stability according to the practitioner (whether the time till next visit should be significantly shorter than the time from the previous visit), 4) mean difference of the IOP (IOP at baseline vs. IOP at the last visit (if at least 24 months afterwards), 5) the results of the examinations and, 6) the number of treatment changes. We did not use glaucomatous progression as an outcome measure, because we did not expect this patient population (with a risk factor for glaucoma or with stable glaucoma) to progress during the study. The change in IOP during the study has been used as outcome measure instead.

### Sample size and power analysis

We performed a post-hoc power analysis using our data to estimate the power (certainty) of our conclusion. We performed that analysis using two outcome parameters since quality of care has multiple dimensions: the stability of the patient according to the practitioner and the overall mark regarding patient satisfaction. The power of the study was > 99% based on the stability outcome when using 5% as an acceptable difference, and > 99% based on the overall mark when using a difference of 0.5 (on a 1-10 scale) as an acceptable difference between the treatment groups as well.

### Patients and visits

**Figure 1:**
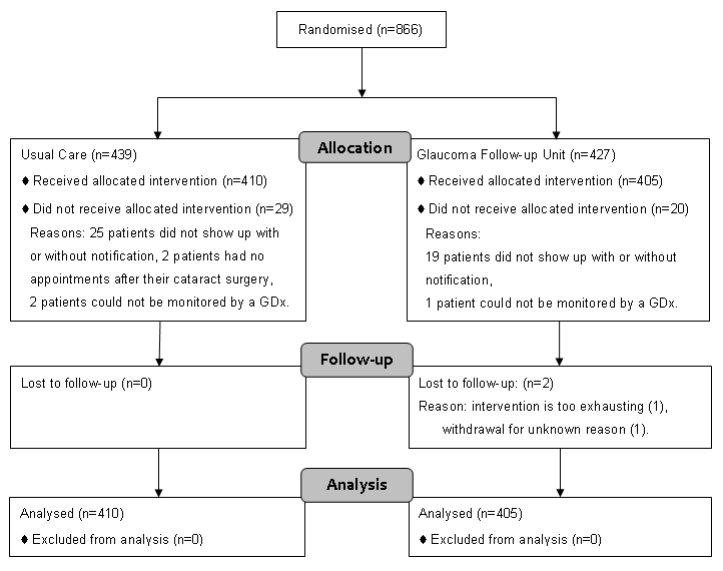
**Patient flow chart**. GDx = Nerve Fiber Analyser.

From September 2005 to March 2006, 866 patients were included of which 46 patients did not visit the hospital during the study period. Three others could not be monitored with the GDx and 2 patients withdrew their informed consent (see Figure 1). The remaining 815 patients had a total of 2100 visits. The average time between visits was 8.8 months, SD ± 4.0. The mean age (63 years) and gender (53% women) was similar for the two treatment groups. There were no significant clinical imbalances between the groups as well (Table 2).

**Table 2:** Characteristics of included patients, by treatment group

	Glaucoma Follow-up Unit (n = 405)		Usual Care (n = 410)	
Gender, % of women	53.8		52.2	
Mean age (SD, standard deviation)	63.0 (12.1)		63.1 (11.9)	
Mean time till next visit in months (SD)	9.8 (2.9)		9.5 (2.9)	
	Right eye	Left eye	Right eye	Left eye
Mean intraocular pressure (SD)	18.7 (4.1)	18.5 (4.1)	18.8 (4.2)	18.8 (4.1)
Mean target intraocular pressure (SD)	25.1 (5.2)	25.2 (5.2)	25.2 (5.4)	25.1 (5.4)

SD = Standard Deviation.

### Study duration

The study duration depended on the allocated treatment group. Patients who were allocated to the usual care group on the day of their visit to the GFU, entered the study at their next visit (to the glaucoma specialist), whereas patients who were allocated to the GFU group, entered the study immediately. Therefore, the mean study duration was longer for the GFU group (1.81 year) than for the usual care group (1.43 year). This difference was statistically significant (p < 0.001). Hence, for a better comparison, we will present the costs per patient year in most tables. The influence of this difference in study duration on the outcomes is probably minimal, because no major changes were made to the protocol over time.

### Economic Evaluation

We conducted an RCT to measure the quality of care delivered by glaucoma specialists and by employees of the GFU. Alongside this RCT, we calculated the costs of glaucoma care from four perspectives. The perspectives used were those of the patient, the REH, the health care system and the society.

A difference in health outcomes between the GFU and the usual care group was not expected during this study, because of the slowly progressive nature of this disease. A literature review, searching for articles with glaucoma and co-management or shared care in the title or abstract, provided evidence of an equal quality of care by optometrists compared to ophthalmologists as well. [3,4,8-18] Only one of the articles reported a variation in individual performances of optometrists, which makes education and accreditation an essential prerequisite for co-management. [10] All other articles reported good quality of care by optometrists, high levels of agreement between optometrists and a research clinic reference or ophthalmologists or comparable inter- and intra-observer variability in optic disc assessments. Therefore we will not present a cost-effectiveness ratio, but we will discuss the costs in relation to the quality of care.

### Identification of cost items and measurement of the utilization per cost item

We interviewed health care professionals and patients to identify relevant cost items in the field of medical consumption, implementation of GFU, and patient time and travel costs.

During the RCT, the medical procedures performed and the medication prescribed each visit were recorded in a case report form. The different types of hospital visits were a visit to: a glaucoma specialist, a resident, and three types of GFU visits, as there were three different types of personnel within the GFU (optometrist, ophthalmic technician level 1, and ophthalmic technician level 2). Per health care provider, the duration of 10 study related visits was measured. The duration of an HFA and GDx test were also measured in 10 patients.

Every visit, glaucoma patients were given a questionnaire to report their travelling distance, mode of transport, travelling time, waiting time and working status, in order to calculate the time and travelling costs. We also examined the fraction of visits in which the GFU employees asked a glaucoma specialist for advice over time. In addition, we performed a logistic regression to determine which variables influenced the probability of asking advice.

The substitution of care to the GFU required organizational changes and hence implementation costs (both initial and structural) within the hospital. To collect this information, health care providers were interviewed.

### Valuation of the cost items

All costs were calculated (in euros, price level 2007) according to the CVZ (The Health Care Insurance Board) costing guidelines and previous research in the REH.

[19] Relevant items from the CVZ costing guidelines [20,21] were updated and used for the calculation of patient time costs per hour and travelling costs per kilometer.

Our cost calculation of hospital costs is based on data from the internal budget allocation provided by the REH financial administration. This information included location costs, costs of medical specialists and other personnel, administrative costs, costs of equipment, overhead costs and interest. Only for the costs of non-laser operations was the DBC rate (Diagnosis Treatment combination- a fixed reimbursement rate for a specific diagnosis related therapy) in 2007 used as estimate of the resource costs.

The direct personnel costs were calculated based on the mean duration for each type of visit. However, the indirect personnel and overhead costs were calculated top-down, based on the mean duration of a visit in the hospital as a whole.

The implementation costs, like internal preparatory meetings, visits to another Dutch hospital, writing the standard working protocol and the training of the employees of the GFU were dominated by personnel input. These costs were added to the costs of a GFU visit as implementation costs for the GFU. The initial implementation costs that were only made before starting the GFU were spread over 5 years. The structural costs per year were added to the initial implementation costs per year. The implementation costs per visit were based on the total number of GFU visits in 2007 (1598 visits) as we expect this number of patients to be a representation of the number of patients in the near future.

We calculated the patient costs using the information of the patient questionnaires combined with the updated time and travelling costs per unit of time and per kilometer. The results will be expressed as average costs per patient per study year and average costs per patient.

### Sensitivity/scenario analysis

To determine the influence of uncertainty regarding the duration of visits or tests on the costs per patient year, we performed the following uni-variate sensitivity analyses:

1. We varied the duration of the visits within the range we had measured in our study. This resulted in 4 scenarios:

a. We used the minimum duration for all visits;

b. We used the maximum duration for all visits;

c. We used the minimum duration of visits to the GFU and the maximum duration for the visits to the glaucoma specialist and resident;

d. We used the maximum duration of visits to the GFU and the minimum duration for the visits to the glaucoma specialist and resident.

2. We used the norm duration of the GDx and HFA as used by the financial department, instead of the duration of the GDx and HFA measured in our study.

Furthermore we performed scenario analyses to determine the effects of plausible policy changes in the (near) future on the costs. We considered the following scenarios:

3. No optometrists are working in the GFU. This actually happened during the course of the study. The direct personnel costs of visits to optometrists were replaced by those of the ophthalmic technicians.

4. In the study, the patients in the GFU group visited the glaucoma specialist (or resident) every third visit, or earlier if necessary. In this scenario, we calculated the visit costs if this routine was changed to every fifth visit (or earlier when necessary). In case of a non stable patient, we distinguished two scenarios:

a. The patient returned to the GFU as soon as he was judged as stable by the glaucoma specialist during a visit.

b. The patient only returned to the GFU after he was judged as stable by the glaucoma specialist on two consecutive visits.

### Uncertainty analysis

We performed a bootstrapping analysis on the costs per patient year and two quality of care parameters, to show the degree of uncertainty regarding the results. Since quality of care has different dimensions, we decided to use two outcome parameters. One clinical quality parameter: stability according to the practitioner (stability), and one patient satisfaction parameter: the overall mark given by the patient. By plotting all bootstrap replicates in a so-called cost-effectiveness plane (CE-plane), the uncertainty around the point estimates of the costs and effects was displayed. In this analysis individual observations of patients were randomly drawn from the distribution of patients in both groups in order to calculate the average costs and quality of care per treatment group. This was replicated for 2500 times. A CE-plane is an x-y-diagram with the x-axis representing the difference in quality of care between the GFU and usual care group and the y-axis representing the difference in costs.

### Statistical analysis

We used Excel for the bootstrapping analysis. SPSS 15.0 was used for all other analyses. In normally distributed variables, we performed a t-test for independent samples. If not distributed normally, we performed the parametric Mann-Whitney U-test to compare the two treatment groups. We used bootstrapping for deriving the 95% confidence intervals around the utilization and costs because of the non-normal distribution of those parameters.

For some visits (29%), information about one or more items related to patient costs was missing. The travelling distance could be calculated for every patient, based on the Zip code as known in the hospital information system. If appropriate, the remaining missing values were replaced by values known from other visits of the same patient. In all other cases (9%), the mean value of a comparable group of patients based on gender and age was imputed to the missing values.

## Results

### Quality of care

The aspects of quality of care measured in our study were: compliance to the protocol, patient satisfaction, stability according to the practitioner, mean difference of the IOP, results of the examinations and the number of treatment changes. All these aspects of the quality of care turned out to be similar for the 2 groups (see also reference 7) and the substitution of care to the GFU was successfully implemented.

1. The GFU employees performed the required tests in at least 98.8% of the visits and referred back to the glaucoma specialist in 84.4% of the remarkable cases.

2. The patient satisfaction was similar in both groups. The overall mark of the patient was 8.5 for the GFU group and 8.4 for the usual care group (p = 0.147).

3. The percentage of visits that were considered "stable" was 16% in the usual care group and 17% in the GFU group (p = 0.423)

4. No statistical difference was found between the two groups in the difference of the IOP during the study (IOP _(≥ 24 months since baseline) _*- IOP *_(at baseline)_).

5. The average difference in IOP OD was -0.2 mmHG in the usual care group and -0.6 mmHG in the GFU treatment group (p = 0.207). The average difference in IOP OS was -0.1 mmHg in both groups (p = 0.915).

6. The number of treatment changes was 57 (14%) in the GFU group and 63 (15%) in the usual care group (p = 0.603).

7. Patients as well as GFU employees and glaucoma specialists were pleased with the functioning of the GFU.

Therefore, the quality of care provided in the GFU was concluded to be equal to the care provided by the glaucoma specialists for these stable glaucoma patients.

### Hospital perspective

The hospital costs covered hospital visits, diagnostic procedures and further treatment, but were mainly driven by the costs of the hospital visits to the glaucoma specialist, resident or GFU employee (approximately 80%). Table 3 shows the duration and composition of the unit costs per type of visit. The total annual implementation costs for starting up the GFU were €4917 for 1598 GFU visits. The implementation costs of the GFU were added to the GFU visits only.

**Table 3:** The composition of the unit costs per type of hospital visit in € (2007)

	Visit glaucoma specialist	Visit resident	Visit GFU Optometrist*	Visit GFU TOA level 1 **	Visit GFU TOA level 2***
**Costs per visit**					
Total direct personnel costs	24.36	14.49	19.09	15.05	16.61
Total indirect personnel costs	5.46	5.46	6.59	6.59	6.59
Total overhead costs	29.76	29.76	35.90	35.90	35.90
Implementation costs GFU	0.00	0.00	3.08	3.08	3.08

**Total costs excluding GDx**	59.58	49.71	64.66	60.62	62.18
Costs GDx**** (fraction performed)	25.00 (0.41)	22.25 (0.36)	3.05	3.05	3.05

**Total costs including GDx**	**84.58**	**71.96**	**67.71**	**63.67**	**65.23**
Mean visit duration (min)	9.06	11.00	20.40	20.40	20.40

*Visit to an optometrist or senior employee

**Visit to an ophthalmic technician level 1

***Visit to an ophthalmic technician level 2

****At the start of the Glaucoma Follow up unit (GFU), a Nerve Fiber Analyser (GDx) was purchased by the Rotterdam Eye Hospital (REH). The costs of the GDx performed during GFU visits, consists only of the GDx imaging device. In the usual care group, the GDx was performed during an extra visit to the perimetry department. In that situation, the costs of a GDx image included personnel and overhead costs as well and were €61.61 based on a duration of 13.30 minutes.

Table 3 shows that despite their longer duration, GFU visits were less expensive than those to the glaucoma specialist. In the usual care group, most visits were paid to the glaucoma specialist or resident. Patients in the GFU group visited the glaucoma specialist every third visit or earlier when a patient was judged not stable. Therefore, the costs per visit could vary within one patient and between patients within one treatment group. The mean costs per hospital visit including GDx were €83.77 (SD = 30.64) in the usual care group and €68.34 (SD = 15.66) in the GFU group. This difference was statistically significant (t-test, p = 0.000).

Table 4 describes the hospital care use per patient year for the two treatment groups. Although the number of visits per patient year was slightly higher in the GFU group (1.65 vs. 1.57), this difference was not statistically significant. In the GFU group, a significantly larger number of GDx images (1.28 vs. 0.77) and auto-refractions (0.20 vs. 0.08) was performed and more time was spent on asking advice (in 24% vs. 10% of the visits). On the other hand, glaucoma surgery, laser therapy, medication use and the number of HFA tests did not statistically differ between the two groups.

**Table 4:** Average hospital care use per patient year for the two treatment groups

	GFU	Usual care	95%-CI of difference between 2 groups	P-value	Costs per unit (in €)
Hospital visits	1.65	1.57	-0.13 to +0.31	0.158	See Table 2
GDx ECC	1.28	0.77	+0.32 to +0.73	0.000	61.61
HFA	0.10	0.11	-0.11 to +0.07	0.266	158.44
Refractive Unit	0.01	0.05	-0.09 to +0.00	0.002	32.43
Auto-refraction	0.20	0.08	-0.03 to +0.21	0.000	4.64-6.59*
Pachymetry	0.02	0.04	-0.07 to +0.03	0.246	23.17
IOP diurnal curve	0.01	0.02	-0.04 to +0.01	0.109	92.66
Laser treatment	0.002	0.007	-0.02 to +0.01	0.267	78.38
Glaucoma surgery	0.002	0.001	-0.01 to +0.01	0.558	1251.70
Asking advice	0.24	0.10	+0.05 to +0.26	0.000	8.19-15.86**
Proportion patients using medication	0.57	0.59	-0.17 to +0.15	0.614	2.53-18.82***

GFU = Glaucoma Follow-up Unit; CI = confidence interval; GDx ECC = Nerve Fiber Analyser Enhanced Corneal Compensation; HFA = Humphrey Field Analyser; IOP = Intra Ocular Pressure.

*Depending on health care provider

**Costs per advice, depending on the health care providers involved

***Costs per month

The hospital care use has been translated into costs per patient year for the two treatment groups in Table 5. The total hospital costs were significantly higher for the usual care group than for the GFU group, mainly because of the higher hospital visit costs. The costs of asking advice were modest, but significantly higher for the GFU group than for the usual care group, as was to be expected. The 95% confidence interval for the difference in total hospital costs as derived from the bootstrap analysis was €-59 to €-2. The probability that the GFU reduces hospital costs was 98%.

**Table 5:** Average costs in Euros per patient year per perspective used for the two treatment groups (SD)

	GFU	Usual Care	P-value
**Hospital perspective**			
Hospital visits (including GDx ECC)	111.93 (50.93)	133.17 (50.44)	0.000
Other tests (HFA, refraction, pachymetry, etc.)	20.66 (47.03)	24.18 (48.72)	0.001
Laser treatment related to glaucoma	0.18 (2.54)	0.57 (5.18)	0.258
Glaucoma surgery	2.84 (40.35)	1.72 (34.90)	0.558
Asking advice	3.24 (5.35)	1.78 (5.13)	0.000
**Total hospital costs per patient year**	**138.85 (89.30)**	**161.43 (86.88)**	**0.000**

**Patient perspective**			
Patient costs per visit			
Travelling costs of patient and accompaniment	8.26 (11.83)	8.19 (12.10)	0.966
Time costs of patient and accompaniment	40.58 (28.87)	47.51 (34.36)	0.000
*Total patient costs per patient per visit*	48.83 (33.68)	55.70 (37.88)	0.000
			
Patient costs per patient year			
Travelling costs of patient and accompaniment	13.04 (17.16)	12.70 (17.87)	0.488
Time costs of patient and accompaniment	66.62 (50.20)	75.17 (61.37)	0.088
**Total patient costs per patient year**	**79.66 (58.51)**	**87.87 (68.17)**	**0.143**

**Health care perspective**			
Hospital costs	138.85 (89.30)	161.43 (86.88)	0.000
Medication costs	91.54 (101.37)	89.82 (100.53)	0.867
**Total health care costs per patient year**	**230.39 (154.57)**	**251.26 (146.02)**	**0.004**

**Societal perspective**			
Hospital costs	138.85 (89.30)	161.43 (86.88)	0.000
Patient costs	79.66 (58.51)	87.87 (68.15)	0.143
Medication costs	91.54 (101.37)	89.82 (100.53)	0.867
**Total societal costs per patient year**	**310.05 (181.86)**	**339.13 (180.39)**	**0.009**

SD = Standard Deviation; GFU = Glaucoma Follow-up Unit; GDx ECC = Nerve Fiber Analyser Enhanced Corneal Compensation; HFA = Humphrey Field Analyser.

The proportion of visits by GFU employees needing advice increased initially from 15% to 20% in 2006 and then decreased (statistically significant) to 13% in 2007 and 7% in 2008. The proportion of visits requiring advice was not affected by the total number of visits per patient.

These findings were confirmed by a logistic regression. The year of the visit was the only variable that significantly influenced the probability of asking advice. The other variables in the regression analysis were: stable/not stable, visit number, gender, time till next visit and age. This indicates that the GFU employees got more experienced over time and therefore needed less advice.

### Patient perspective

The patient costs consisted of time and travelling costs of patients and their accompaniment. Table 5 shows that the patient costs per visit were significantly higher in the usual care group, because of higher time costs (€80 vs. €88). This was mainly caused by a longer waiting time in the hospital in the usual care group. Patients in the GFU group spent, on average, 44.6 minutes in the hospital against 59.4 minutes for the patients in usual care group (p = 0.000). However, because of a higher number of visits per patient year in the GFU arm, the patient costs per patient year were not statistically significantly higher anymore. The 95% confidence interval based on the bootstrapping analysis confirmed this (€-29 to €12). However, there is still a 78% probability to reduce patient costs.

### Health care perspective

The health care costs consisted of the hospital costs as described above, and of medication costs. Table 5 shows the health care costs per patient year. Because of the comparable medication costs and lower hospital costs in the GFU group, the total health care costs per patient year were nearly 10% lower for the GFU group (€230.39 vs. €251.26, p = 0.04). The median cost differ statistically according to the Mann-Whitney U-test, but the confidence interval as provided through bootstrapping does not show a difference in the mean costs (€-76 to €21). However, the probability of cost reduction is considerable: 87%.

### Societal perspective

In the societal perspective all costs were taken into account. It consisted of hospital costs, medication and patient costs, for 46%, 28% and 26% respectively. The total societal costs per patient year were almost 10% higher in the usual care group (Table 5: €339.13 vs. €310.05, p = 0.009). The mean difference in the total societal costs per patient year was €-36 (the GFU group was less expensive). The 95% confidence interval based on bootstrapping for this difference ranged from €-92 to €23. Thus, though the median costs per patient year differs between the two groups, the mean total costs are not statistically different. This is because the non-normal distribution of the societal costs. However, the probability that the GFU saves societal costs is 84% to 89% (see paragraph about the uncertainty analysis below).

### Sensitivity/scenario analysis

#### Analysis 1: duration of visit

The mean duration of the visits to the glaucoma specialist was 9 minutes (ranging from 7 to 11 minutes), to the resident 11 minutes (ranging from 9 to 13 minutes) and to the GFU 20 minutes (ranging from 16 to 24 minutes).

In the base case - the situation as in our study -, the GFU group was less expensive than the usual care group. This conclusion only changed when the duration of a visit in the usual care group would be relatively short (7 minutes) and the duration of a visit in the GFU group would be relatively long (24 minutes, scenario 1d). In that unlikely situation the hospital costs per patient year were 10% higher for the GFU group (see Table 6).

**Table 6:** Total average hospital costs per patient year in Euros for all situations in the sensitivity/scenario analysis

	Hospital costs	
	GFU	Usual care
Base case	138.85	161.43
Scenario 1a	117.79	141.80
Scenario 1b	156.79	179.98
Scenario 1c	117.79	179.98
Scenario 1d	156.79	141.80
Scenario 2	153.78	173.18
Scenario 3	135.16	159.47

#### Analysis 2: duration HFA/GDX

The norm durations of GDx and HFA as used by the financial department, were 15 and 45 minutes respectively instead of 13.30 and 34.20 minutes. This longer duration of the HFA and GDx tests increased the hospital costs per patient year in the GFU group and usual care group with €15 and €12 respectively (Table 6).

#### Analysis 3: no optometrist in GFU

When the direct personnel costs of the optometrist were replaced by those of the ophthalmic technicians, the costs in both groups decreased, because incidentally a visit was paid to an optometrist in the usual care group as well (Table 6). Although the decrease in costs per patient year is small, it is almost twice as high in the GFU group (€3.69) as in the usual care group (€1.96). Thus, the costs remain lower for the GFU group.

#### Analysis 4: fewer specialist visits in GFU

a. In this scenario, the total savings were €2193 for five visits of 427 patients. Based on a mean number of 1.65 visits per year as measured in this study, the hospital costs could be reduced with €1.69 per patient year.

b. In the second scenario, the total savings in visit costs were €1882 for five visits of 427 patients, thereby reducing the hospital costs in the GFU group with €1.45 per patient year (= 1%).

### Uncertainty analysis

**Figure 2:**
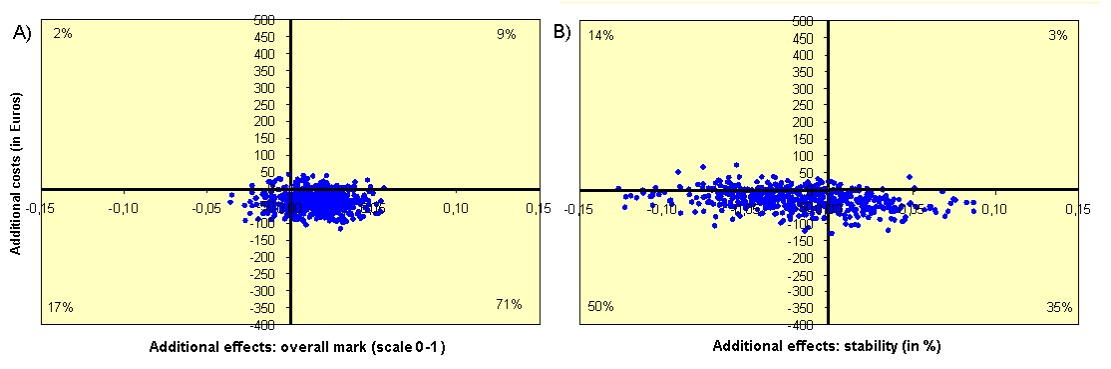
**Cost-effectiveness plane**. A) yearly incremental costs per patient vs. change in the patients' overall mark. B) yearly incremental costs per patient vs. change in the percentage of stable visits.

From a societal perspective, the incremental cost-effectiveness ratio of the GFU compared with usual care was - €27 per patient per decimal point increase of the patients' overall mark (on a 1-10 scale) per year. The CE-plane with overall mark as outcome showed that the majority of bootstrap replications (70%) fell within the lower-right quadrant, indicating that the GFU was dominant with lower costs and a higher overall mark (Figure 2 a).

The incremental cost-effectiveness ratio for the GFU compared with usual care was + €19 per patient per year for one extra percent of visits that were considered to be stable by the practitioner. For the CE-plane with "stability" as outcome, the majority of bootstrap replications fell within the lower-left quadrant which reflects lower costs and fewer stable visits (Figure 2 b). The probability that the GFU is cost saving is 89% using the overall mark and 84% using the "stability" outcome. Against this high probability of saving costs, the probability of inferiority of the GFU (being more expensive and less effective) is quite small: 2% using the overall mark and 14% using the "stability" outcome.

Using an acceptable difference of 0.5 point of the overall mark (range 1-10), and of 5% difference in the fraction of stable patients, the two groups have an equal quality of care in 99.5% and 80.5% of the bootstrap replications respectively. When including replications that result in a better quality of life for the GFU, the quality of care is acceptable (equal or better) in 100% and in 83.6% of the bootstrap replications.

## Discussion

Substitution of tasks that require less specialized skills is a possible solution for easing the increased workload of ophthalmologists and long waiting lists in ophthalmic care. It was hypothesized that task substitution reduces the costs as well. The monitoring of most stable glaucoma patients probably does not require specialized skills. In this study, we therefore compared the care as usual provided by glaucoma specialists with the care provided by a GFU within the REH staffed by ophthalmic technicians and optometrists for stable glaucoma patients.

We found about 10% lower health care costs per patient year for the GFU group compared to the usual care group for three of the four perspectives used: the REH, the health care system and the society. Patient costs did not differ between the two treatment groups.

Scenario and sensitivity analyses confirmed that our results were robust. Only if the mean duration of a visit increased in the GFU (with 18% to the maximum duration measured in this study) and decreased for the glaucoma specialist (with 23% to the minimum duration measured in our study, scenario 1d), would the total societal costs not be significantly different any longer. However, this situation is not realistic. The bootstrap analysis showed that the equivalence of the two groups on quality of care is justified and that the GFU is cost saving in 89% of the bootstrap replicates when using the overall mark as outcome parameter and in 84% of the replicates when using the stability of the patient according to the practitioner.

We hypothesized that the establishment of the GFU would reduce the waiting list. This was confirmed by the increased number of patients (+23%) and patient visits (+16%) per year within the study period. The increased number of visits was largely caused by the establishment of the GFU, whereas the rise in the number of glaucoma patients was also influenced a little by a reduced follow-up interval for some glaucoma patients. However, the long term effect on the waiting list seems to be limited. Possible causes are: the chronic character of the disease which limits the patient outflow and the substantial increase in new glaucoma patients that outweighs the growth in capacity. Further research would be necessary to explore the true cause(s).

The hospital perspective was one of the perspectives used for the cost calculation. Although the probability that the GFU is cost-effective from this perspective is 94-98%, we have to distinguish at least two stakeholders within the hospital; the hospital management and the glaucoma specialists. The interests of those two stakeholders are partially conflicting due to the current structure of financing care in the Netherlands. The physician part of the reimbursement is now paid to the specialist although the monitoring is partially transferred to the GFU. The distribution of this fee will therefore become subject of discussion between glaucoma specialists and the hospital management, especially when health care insurers insist on a lower fee in future negotiations, because of the lower costs of monitoring glaucoma patients by the GFU.

Our results could not be easily compared with results of other research. Even though substituting tasks within the hospital setting is taking place, a full cost calculation of this kind of substitution in the ophthalmic care has not been performed yet. In Bristol (UK), an economic evaluation alongside an RCT has been performed, comparing costs of monitoring stable glaucoma patients by ophthalmologists and community optometrists (outside the hospital). [2,6] Contrary to ours, the UK study concluded that the substitution of care to community optometrists was not likely to save costs. The main reason for this was the larger number of referrals to the ophthalmologist in their study compared to ours (19% vs. 6%). An explanation for this difference might be the location of care. Community optometrists do not have the possibility to consult a specialist for quick advice and will therefore refer patients to the hospital relatively more often.

Furthermore, the frequency of visits to the community optometrist in Bristol was 66% higher than the visit frequency to the ophthalmologist, compared with a 5% higher frequency in our study. This difference is related to a difference in the protocol used. In our study, the time to the next visit was copied from the last visit to the glaucoma specialist instead of being pre-determined at 6 months.

A study about the trends in outpatient care provided by physicians and non-physician clinicians showed that substitution of care is not always a good strategy for containing health care costs. [22] The increase in the proportion of patients visiting a non-physician clinician is driven by the increase in patients visiting both a non-physician and a physician clinician. In our study however, the number of extra visits caused by referrals was relatively low as stated earlier.

A possible drawback of our study is the lack of information about disease progression. The progression rate of glaucoma depends on the intraocular pressure, and the time to vision loss varies between 3 years for untreated patients with a high intraocular pressure to 38 years for well treated patients. [23,24] We therefore did not expect to detect any significant glaucomatous progression in the 30-months study period in these stable patients and performed a cost minimization study. This type of economic evaluation assumes an equal outcome for all patients. The results of the RCT [7] as well as many other studies [3,4,8-18] supported this assumption about the equal quality of care to glaucoma patients provided by different types of health care providers.

## Conclusions

Considering the equal quality of care in both treatment groups, we conclude that monitoring of glaucoma patients by the GFU is cost-effective for a subset of glaucoma patients, i.e., those that were deemed stable in the Rotterdam Eye Hospital. Implementation of a similar GFU in other hospitals could therefore be considered.

## Competing interests

The authors declare that they have no competing interests.

## Authors' contributions

KMHG carried out the cost calculations and drafted the manuscript. EvS surveyed the resource use of the patients, and drafted the manuscript. HGL was involved in setting up the Glaucoma Follow-up Unit and reviewed the manuscript. TP and NSK participated in the design of the study and reviewed the manuscript. MAK participated in the design and coordination of the study and helped to draft the manuscript. All authors interpreted the results, read and approved the final manuscript.

## Pre-publication history

The pre-publication history for this paper can be accessed here:

http://www.biomedcentral.com/1472-6963/10/312/prepub
